# The incidentally diagnosed adult congenital heart disease during routine medical health checkups in 27,897 Koreans at a single center over seven years

**DOI:** 10.1186/s12872-018-0968-0

**Published:** 2018-12-05

**Authors:** Eun Min Kwag, Ju Suk Lee, Sung Hoon Kim

**Affiliations:** Department of Pediatrics, Samsung Changwon Hospital, Sungkyunkwan University School of Medicine, 158, Paryong-ro, Masanhoewon-gu, Changwon, Gyeongsangnam-do 51353 Republic of Korea

**Keywords:** Adult, Congenital heart disease, Echocardiography, General health checkups

## Abstract

**Background:**

The rate of incidentally diagnosed congenital heart disease (CHD) in adulthood has not been reported. The aim of this study was to investigate the detection rate of CHD in adults by routine, general health checkups.

**Methods:**

Data was acquired from 222,401 patients older than 19 years who participated in general health checkups from January 2010 to December 2016. We excluded persons who did not undergo echocardiography during the general health checkups, who underwent echocardiography prior to the health checkups, and who were previously diagnosed with CHD.

**Results:**

Among the 27,897 patients, who were included in the final analysis, 293 cases were newly diagnosed as CHD, and the overall detection rate was 1.05%. The mean age of patients with CHD was 48.7 ± 21.5 years, and most of them were female (*n* = 187, 63.8%). More than two-thirds were between the third and fifth decade of life, and only six patients (2.04%) were older than 70 years. The most common type was bicuspid aortic valve (*n* = 155). Interestingly, Ebstein’s anomaly that required surgical repair was detected in five persons.

**Conclusions:**

During general health checkup, there were cases of severe CHD that required cardiac surgery upon diagnosis.

## Background

Congenital heart disease (CHD) is a term used to describe a wide spectrum of heart diseases that have been present since the time of birth. Recently the live birth incidence of CHD has been reported to be approximately 1% or less [1–5]. According to recent advances in diagnostic tools, such as echocardiography, and cardiac computerized tomography, most cases of CHD can be detected and managed in early life [3]. Consequently, more patients with CHD reach adulthood as therapy becomes increasingly effective and this creates a completely new and steadily growing patient population: patients with grown-up congenital heart disease (GUCH), for example, with estimates of at least 1.2 million cases of repaired CHDs in Europe alone [1, 6]. However, some cases of CHD are not diagnosed during childhood, but later in adulthood. Nowadays, active health management at the prevention level is required for the promotion of health in Koreans, considering the increasing diversification of diseases, falling birth rates, and aging society. Based on this consideration, the National Health Insurance Service (NHIS) actively promotes health checkups in an effort to detect diseases early and enhance public health accordingly. Among the general health checkup tests (whose details are explained in the methods section), echocardiography is not routine, but is a self-selected test paying out of one’s pocket. During checkups, previously undetected CHDs have been diagnosed in adults according to our hospital’s experience. The first aim of this study is to determine the detection rate of newly diagnosed CHD in adults during health checkups. We also wanted to investigate the severity of these incidentally diagnosed CHD by assessing the degree of symptoms and whether the patient opted to have treatment.

## Methods

### Study subjects

This study was based on data obtained from January 1, 2010 to December 31, 2016. Data were collected from people who underwent a general health checkup under the guidance of the NHIS at Samsung Changwon Hospital. Before test, physical examination by doctor was not performed. General health checkups were composed of a variety of diagnostic tests, including interviews about lifestyle whose format was designed by the NHIS Institutional Review Board. The surveys included well-established questions to determine the demographic and socioeconomic characteristics of the participants. These questions were about sex, age, marital status, employment status, education level, residence area, past and present medical history, regarding any type of cancer, alcohol consumption status (frequency and amounts per week), smoking status (never, former, or current smokers), daily number of cigarette smoked, and level of vigorous exercise (≥3 times per week) were also evaluated with standard questionnaires. And physical measurements (height, weight, waist, and body mass index), blood pressure check, visual and auditory acuity tests, chest X-ray, electrocardiography (ECG), laboratory tests (complete blood count and levels of creatinine, glucose, cholesterol, triglyceride, alanine aminotransferase, aspartate aminotransferase, and gamma-glutamyl transferase), dental inspection, and urinalysis were routinely performed for all participants. Pre-hypertension was defined as systolic pressure is between 120 and 129 mmHg and diastolic pressure less than 80 mmHg and hypertension was defined as a condition wherein a person had elevated blood pressure (systolic pressure ≥ 130 mmHg or diastolic pressure ≥ 80 mmHg) according to the 2017 guideline of American College of Cardiology [7]. General health checkups were 100% paid for by the corporation. Additionally, several tests, including echocardiography, were performed for persons who wanted to pay out of pocket. These general health checkups for asymptomatic adults are unique medical phenomenon of South Korea under the NHIS compared to other countries.

We excluded persons who did not undergo echocardiography, those who had undergone echocardiography prior to the checkup, those who were previously diagnosed with CHD including those treated with either cardiac surgery or percutaneous intervention, and those who were younger than 19 years. Usually adult is defined as a person older than 18 years old. However, in our hospital, a person older than 19 years old had performed general health checkups under the NHIS. A person under 18 years old had general health checkups, which was not supported by NHIS, for specific purpose such as certification of immigration or overseas education. Therefore, we used as a cut-off for age as older than 19 years old. Because conditions such as persistent left super vena cava, hypertrophic or dilated cardiomyopathy, and congenital arrhythmias such as long QT and Wolff-Parkinson-White syndrome are not regarded as CHD [1], these diseases were excluded in this study. Although the uncomplicated bicuspid aortic valve (BAV) is known to be the most common CHD affecting 1.3% of the population, BAV is included in this study because we want to investigate the detection rate of the incidentally diagnosed adult CHD. When CHD was found in a later echocardiogram in patients who underwent multiple echocardiographic studies during the study period, only the first echocardiography which diagnosed new CHD was included. Each patient underwent a transthoracic echocardiographic examination with two-dimensional, M-mode, Doppler, and tissue Doppler imaging by sonographers, and the findings were confirmed by attending staff before the final diagnosis was made. For definition, we preferred the detection rate to the incidence or prevalence because of character of study design.

### Statistical analyses

Clinical and echocardiographic data were presented using descriptive statistics, including mean, standard deviation, range, median, minimum and maximum, and percentage. Statistical analysis was conducted using the Statistical Package for the Social Sciences version 21.0 (SPSS Inc., Chicago, IL, USA). For all analyses, *p*-values were two-tailed, and a *p*-value < 0.05 was considered statistically significant.

## Results

### Patient’s characteristics

**Fig. 1:**
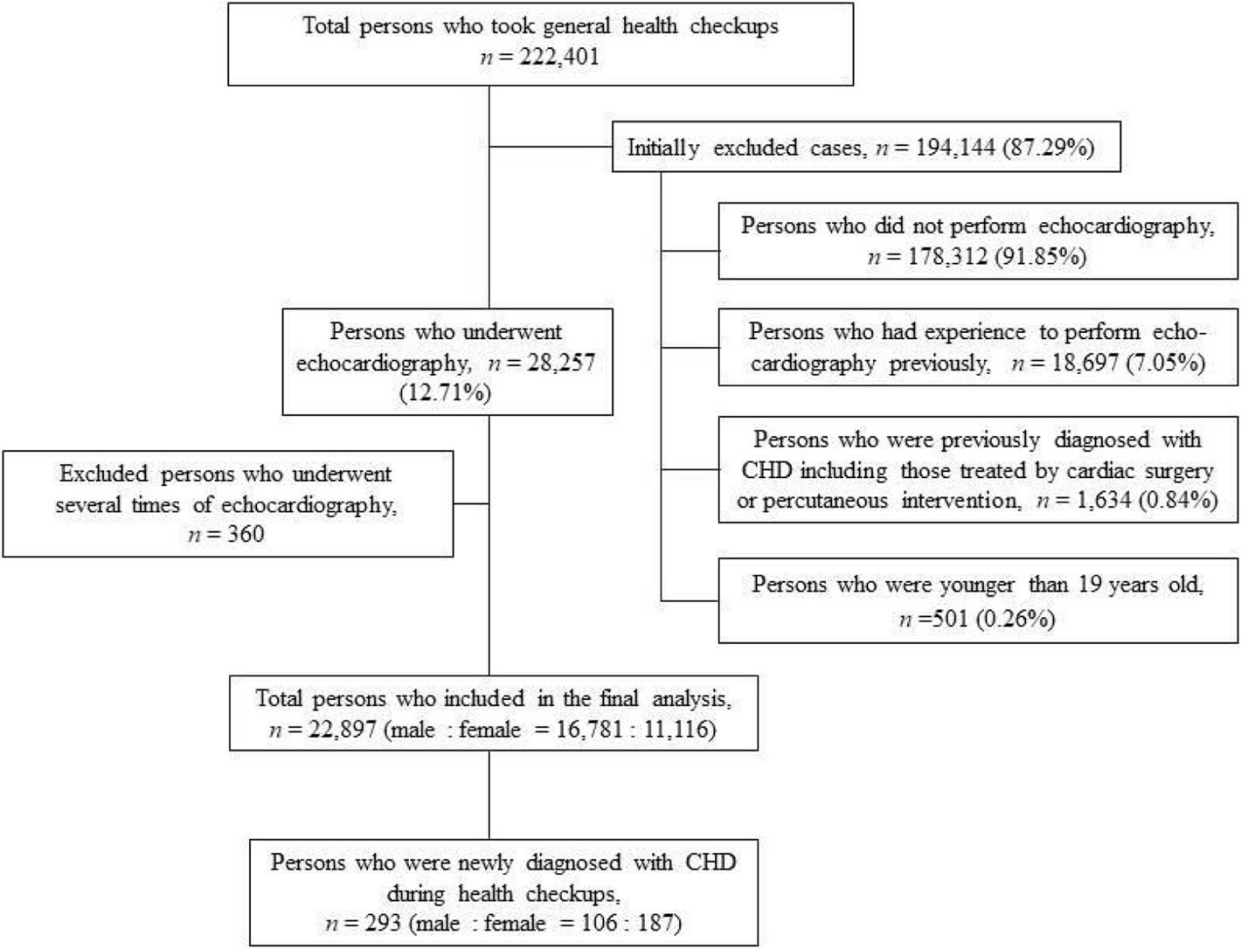
The evaluation flow chart for persons who were newly diagnosed with CHD during general health checkups. CHD; congenital heart disease

Figure 1 reveals the flow chart of diagnosis for persons who were newly diagnosed with CHD during health checkups. There were finally 27,897 (male: *n* = 16,781, female: *n* = 11,116) adult cases who underwent echocardiography for the first time in their life without any recommendation from doctor. For reference, the number of undiagnosed CHD by first test was 13 and they were all atrial septal defects (ASD) patients. Among these, including delayed diagnosed 13 patients, 293 cases were newly diagnosed as CHD in the first time in their life, and the overall detection rate was 1.05%. Table 1 shows the detection rate of new adult cases of CHD by study year, and Tables 2 and 3 shows their demographic characteristics and disease distribution, respectively. The mean age of patients with CHD was 48.7 ± 21.5 years, and most of them were female (*n* = 187, 63.8%). About more than two-thirds (*n* = 241, 82.3%) were between the third and the sixth decade of life, and only six patients (2.04%) were older than 70 years. The youngest and oldest patients reported in this study were women with ASDs who were 21 and 79 years old, respectively.

**Table 1:** Yearly detection rate of congenital heart disease (CHD)

Year	Performance of echocardiography (*n* = cases)	Diagnosis of CHD (*n* = cases)	Detection rate (%)
2010	3073	26	0.85%
2011	4192	39	0.93%
2012	4248	36	0.85%
2013	4101	54	1.32%
2014	4208	48	1.14%
2015	4142	51	1.23%
2016	3933	39	0.99%
Total	27,897	293	1.05%

**Table 2:** Demographic characteristics of newly diagnosed congenital heart disease subjects

Numbers (Proportion, %)	
Total numbers	293
Sex	
Male	106 (36.2%)
Female	187 (63.8%)
Age (years, male: female) (proportion, %)	
20–30	5 (3:2) (1.7%)
31–40	79 (36:63) (27.0%)
41–50	83 (39:54) (28.3%)
51–60	86 (39:52) (29.4%)
61–70	33 (18:23) (11.3%)
> 70	6 (1:5) (2.0%)
Marital status	
Married	213 (72.7%)
Single (separated or divorced)	12 (4.1%)
Never married	68 (23.2%)
Employment status	
Employed	197 (67.2%)
Unemployed	96 (32.7%)
Education	
< High school	12 (4.1%)
High school	157 (53.6%)
> High school	124 (42.3%)
Residence area	
Urban	227 (77.5%)
Rural	66 (22.5%)
Self-reported smoking status	
Non-smoker	125 (42.7%)
Ex-smoker	51 (17.4%)
Current-smoker	117 (39.9%)
Body mass index	
< 23	91 (31.1%)
23–25	86 (29.4%)
≥ 25	116 (39.6%)
Hypertension	
No	159 (54.3%)
Pre-hypertension	84 (28.7%)
Hypertension	50 (17.1%)

Pre-hypertension is defined as systolic pressure is between 120 and 129 mmHg and diastolic pressure less than 80 mmHg according to the 2017 guideline of American College of Cardiology

**Table 3:** Distribution of newly diagnosed congenital heart diseases (CHD, *n* = 293 cases)

	Numbers of CHD (%)	Male/Female (*n* = cases)
Bicuspid aortic valve	155(52.9%)	106/49
Atrial septal defect	102 (34.8%)	27/75
Ventricular septal defect	14 (4.78%)	9/5
Pulmonary stenosis	12 (4.09%)	5/7
Patent ductus arteriosus	8 (2.73%)	6/2
Coarctation of Aorta	3 (1.02%)	2/1
Partial anomalous of pulmonary venous returns	2 (0.68%)	1/1
Cor triatriatum	7 (2.39%)	3/4
Ebstein’s anomaly	5 (1.71%)	1/4
Congenitally corrected transposition of great arteries	2 (0.68%)	0/2

In the interview of 293 patients, the reported symptoms of dyspnea, cyanosis, fatigue, or chest tightness related to CHD, had the following frequencies; 1.29, 0, 5.81, and 0.65%, respectively. In contrast, those symptoms were found in the rest 222,108 cases following frequencies; 0.25, 0, 3.13, and 0.11%. All participants attending general health checkups routinely took an ECG and among them, if any type of arrhythmia was detected they were recommended to visit the cardiology outpatient clinic. Twenty-one patients described intermittent palpitation during daily activity and there were several arrhythmias such as first degree atrioventricular block (*n* = 1), premature atrial contracture (*n* = 8), and premature ventricular contracture (*n* = 4) on resting ECG during general health checkups. Seven of them performed 24 h ambulatory ECG monitoring (Holter test), only two cases were found that they have atrial fibrillation. The mean cardiothoracic ratio of patients with CHD on chest X-ray was 0.54 ± 0.25, in contrast to 0.48 ± 0.54 in rest 27,604 cases without CHD (*p* = 0.769). The most common type of CHD was BAV (*n* = 155, 52.9%) and the detection rate of it (155/27,897 = 0.56%) was relatively lower than the incidence of it in the general population (1.3%) [1, 8]. Similarly it was more male predominance (*n* = 106, 106/155, 68.4%) [1, 8]. Isolated left-to right shunt and obstructive lesions included ASD (*n* = 102, 34.8%), ventricular septal defects (VSD, *n* = 14, 4.78%), pulmonary stenosis (PS, *n* = 12, 4.09%), and patent ductus arteriosus (PDA, *n* = 8, 2.73%). Except BAV, these four simple lesions represented the majority of newly diagnosed adult CHD cases (46.4%). No cases of cyanotic heart disease, including Tetralogy of Fallot (ToF) or Eisenmenger syndrome, were reported. We next briefly describe the clinical characteristics and outcome of ASD, VSD and Ebstein’s anomaly.

### Atrial septal defect (ASD)

ASD was reported in was more common in females (*n* = 75, 73.5%) than in males. The average size of the defect was 22.1 ± 6.3 mm in the parasternal short axis view. Transesophageal echocardiography was performed in 38 cases (37.26%) for confirmation of the defect and to check the type or size of the defect. The secundum type was the most common type (*n* = 89), followed by the primum type, (*n* = 7), and the sinus venosus type (*n* = 6). No cases of coronary sinus ASD were reported in this study, and treatment was performed for a total of 46 cases (cardiac surgery, *n* = 27; intervention with device closure, *n* = 19).

### Ventricular septal defect (VSD)

The mean age of these patients was 34.7 ± 15.7 years (median: 31.9 years), which was younger than those with ASD (48.6 ± 14.5 years, *p* = 0.02). Most lesions were small defects, with an average size of 0.78 ± 0.67 mm. The most common type was perimembranous (*n* = 7) and subarterial type was the second common (*n* = 5), and the other cases were muscular type. Cardiac surgery was performed in four patients during outpatient clinic follow-up due to progressive moderate aortic regurgitation (AR) in cases of subarterial VSD (*n* = 3) and volume overloading in a case of perimembranous VSD (*n* = 1).

### Ebstein’s anomaly

The mean age of these patients was 56.7 ± 13.4 years (median: 61.7 years) and mainly were female. Although most patients demonstrated symptoms, such as fatigue and palpitation, definitive cardiomegaly on chest x-ray (cardiothoracic ratio: 0.61 ± 0.23), which was statistically significant when compared to those of all other CHD patients, 0.51 ± 0.27, *p* < 0.01), and moderate tricuspid regurgitation on echocardiography. Among them, three underwent cardiac surgery, but the remaining two refused surgery and were lost to follow-up.

## Discussion

We reported a study population-based detection rate of incidentally diagnosed adult CHD. It is of interest to know how many individuals who have their CHD first detected in adult life. The present study reported the 7-year detection rate, type distribution, and outcomes of CHD in adult Koreans, especially those diagnosed during general health checkups.

There are many reports related to GUCH patients with or without repair [1, 9, 10], however, some adults with CHD are asymptomatic or have nonspecific findings during physical examination, leading to a portion of affected adults being undiagnosed. And there are relatively few studies about newly diagnosed CHD in adulthood [3], and they usually report the incidence or prevalence of adult CHD cases diagnosed in clinical practice, but do not include those diagnosed during health checkups [2, 3]. We think the true incidence of newly diagnosed adult CHD is close to the sum of the number of cases diagnosed in outpatient clinics and those diagnosed during health checkups. Therefore, we investigated the detection rate of previously undetected, unrepaired adult CHD through general health checkups, showing a result of 1.05% (293/27,897). Because we cannot find any literature reported that it would be high if over 0.5% of relatively asymptomatic patients with normal exams have CHD [1–5, 11], we cannot conclude whether this detection rate is high or low. A multicenter collaborative study is needed for more accurate determination.

Our study showed a female preponderance (63.8%), which agreed with a previous report stating that the prevalence of CHD in adults was significantly higher in females than in males (4.55 per 1000 females vs. 3.61 per 1000 males; *p* < 0.0001) [2]. On the other hand, there have been some reports showing no gender difference among adult patients with CHD [6, 12]. The inconsistent findings on gender differences among studies are possibly due to the different geographical areas, racial differences, and other differences in study populations and study design. However, previous results showed an overall gender difference in GUCH patients, not specifically in newly diagnosed adult CHD, as in our study. Although the top five types of GUCH patients are known to be ASD, VSD, PDA, ToF, and PS [1–3, 6, 13], because our study excluded previously diagnosed or repaired GUCH patients, cases of ToF were not reported. In concordance with the results from other reports related to GUCH patients, ASD was the most common type of CHD except BAV that was newly diagnosed in adulthood through general health checkups. Small ASDs are typically asymptomatic with insignificant findings during childhood. Therefore, most patients with ASD survive into adulthood without being diagnosed with CHD, which leads to it having the highest incidence among newly diagnosed adult CHD cases.

Undetected VSDs in adults are almost always small defects that place little hemodynamic load on the heart and pose no risk of pulmonary vascular disease [1]. However, according to a previous report, although 87% of patients with subarterial VSD did not have AR at the time of diagnosis, 46% developed AR during an average follow-up of 8 years [14]. Surgery must be considered when AR is detected [1]. Similarly, small subarterial VSDs had a relatively high incidence in our study (*n* = 5/14, 35.7% of total VSD), and surgery was performed during outpatient clinic follow-up in three patients due to progressive AR. Compared to ASD, VSD and PS can be easily detected during physical examination due to its cardiac murmur. But in our study, patients with VSD and PS were not diagnosed before general health checkups. We hypothesized that this fact may be related to silent murmur in especially subarterial VSD or inattention during physical examination by prejudice of doctor who has the tendency to think like that adult without any symptoms has no CHD. If anyone was recommended to perform echocardiography by doctor due to cardiac murmur or any suspicious symptoms of CHD, this may be a bias in this study. But in our study, all participants, who underwent echocardiography to pay out of pocket, made a choice of that procedure among several self-selected tests.

Interestingly, we thought the incidence of Ebstein’s anomaly might be also low in this study as the detection rate being 0.018% (5/27,897) but also be considered high as 1.71% among the incidentally detected CHD (5/293). Because it is a rare congenital cardiac disease occurring in 1 per 200,000 live births and accounting for < 1% of all CHD and in the natural history of this congenital disease, only 5% of patients survive beyond the fifth decade [15, 16]. If the deformity of the tricuspid valve is severe, it can result in progressive congestive heart failure, cyanosis, or even in death during the neonatal period. At the other end, patients with a mild degree of tricuspid displacement can remain asymptomatic until late adult life [15]. In our study, although five persons with Ebstein’s anomaly had either symptoms or definite cardiomegaly in X-rays in order to be referred for cardiac surgery when they were diagnosed, anyone had not taken a medical examination by doctor before general health checkups. Since they were all missed diagnosis, we emphasize that it would be better to educate clinicians to recognize that there might be undiagnosed CHDs in the clinical practice.

This study had several limitations. This is a population-based study, however, the population screened might not be generalizable to all adults in South Korea. And in the point of that ASD and partial anomalous pulmonary venous return could be easily missed initially for beginner during echocardiography, there might be other patients with ASD and partial anomalous pulmonary venous return among them who were revealed to have no CHD during our general health checkups. For example, in our study, the number of undiagnosed CHD by first test was 13 and they were all ASD. And BAV is the most common CHD with more male predominance, but we included it in this study. Therefore, if we had excluded BAV in this study, the detection rate, age, and gender difference would be changed. Also, we analyzed the results of adults who underwent echocardiography only during general health checkups. In the perspective of other countries, regular health check-ups, once or twice annually, of asymptomatic people (without even a family history of a disease with possible genetic disposition) in the middle of their life, seem to be unjustified. However, as previously mentioned, this phenomenon is a unique medical situation in South Korea. And also echocardiography was only performed in a minority of individuals (28,257/222,401, [12.71%]) who wanted to do it for oneself because it must be paid in order to perform. This will be a significant bias. Therefore, our results might not reflect the true incidence of newly diagnosed CHD in adulthood. A large population-based study or multicenter collaborative study is needed for more accurate determination of the incidence rate of newly diagnosed adult CHD**.** However, to the best of our knowledge, this study is the first to document the detection of newly diagnosed adult CHD using general health checkups.

## Conclusion

This population-based study yielded new information on the detection rate of newly diagnosed adult CHD between 2010 and 2016 during general health checkups. In this study, there were some adults without any symptoms related to CHD, however, there were also cases of severe CHD that required cardiac surgery upon diagnosis, like those with Ebstein’s anomaly. There was a female predominance among adults with CHD, and ASD was the most common type of CHD except BAV.

## Abbreviations

ASD: Atrial septal defects
BAV: Bicuspid aortitc valve
CHD: Congenital heart disease
GUCH: Grown-up congenital heart disease
NHIS: National Health Insurance Service
PDA: Patent ductus arteriosus
PS: Pulmonary stenosis
ToF: Tetralogy of Fallot
VSD: Ventricular septal defect
